# Neural network hyperparameter optimization for prediction of real estate prices in Helsinki

**DOI:** 10.7717/peerj-cs.444

**Published:** 2021-04-19

**Authors:** Jussi Kalliola, Jurgita Kapočiūtė-Dzikienė, Robertas Damaševičius

**Affiliations:** 1 Department of Applied Informatics, Vytautas Magnus University, Kaunas, Lithuania; 2 Faculty of Applied Mathematics, Silesian University of Technology, Gliwice, Poland

**Keywords:** Artificial neural network, Hyperparameter optimisation, Prediction model, Real estate prices

## Abstract

Accurate price evaluation of real estate is beneficial for many parties involved in real estate business such as real estate companies, property owners, investors, banks, and financial institutes. Artificial Neural Networks (ANNs) have shown promising results in real estate price evaluation. However, the performance of ANNs greatly depends upon the settings of their hyperparameters. In this paper, we apply and optimize an ANN model for real estate price prediction in Helsinki, Finland. Optimization of the model is performed by fine-tuning hyper-parameters (such as activation functions, optimization algorithms, etc.) of the ANN architecture for higher accuracy using the Bayesian optimization algorithm. The results are evaluated using a variety of metrics (RMSE, MAE, R2) as well as illustrated graphically. The empirical analysis of the results shows that model optimization improved the performance on all metrics (reaching the relative mean error of 8.3%).

## Introduction

Artificial Intelligence (AI) and Machine Learning (ML) have been implemented into many industrial and business fields and applications (Cioffi et al., 2020; Kraus, Feuerriegel & Oztekin, 2020). Influential studies have demonstrated the robustness of the AI/ML approaches to predict (or classify) different factors (as interest rates, mortgage rates, prices, etc.) in the real-estate sector (Mu, Wu & Zhang, 2014; Kang et al., 2020). The real estate price prediction problem is one of the most popular topics in which the capabilities of AI-ML are investigated. Besides, real estate price prediction is a complex non-linear problem, which is affected by multiple direct and indirect attributes (such as construction year, apartment area, etc.) (Ferlan, Bastič & Pšunder, 2017).

Different types of approaches are used to predict the prices of residential property. One of the methods most popular methods is based on hedonic price models (Can, 1992). Hedonic price models (HPM) are relatively easy to analyze and simple to implement. Besides, HPM allows human intervention to produce different outcomes consistently; and due to it, developers can have a better understanding of relationships between inputs and outputs (Tajani, Morano & Ntalianis, 2018). Despite the advantages of HPM, they suffer from the non-linearities problem which is common in the price prediction tasks. The linear approach for real estate price prediction has difficulties to construct generic predictions, therefore other approaches have been offered to cope with the non-linearity problem.

On the contrary to the hedonic models, other more sophisticated price prediction approaches focus on the non-linear relationship between input and output data such as spatial lag and spatial error model (Wang, Chang & Wang, 2019). The classic representatives of this group are regression methods, such as linear regression, support vector regression (SVR) or k-nearest neighbors, and random tree regression for real estate price predictions are widely used and have demonstrated satisfactory results in the past (Madhuri, Anuradha & Pujitha, 2019; Manasa, Gupta & Narahari, 2020; Lu et al., 2017; Hong, Choi & Kim, 2020). However, regression methods have achieved only slightly better results compared to HPMs, but they are more complicated and expensive to set up.

As an alternative to classic price prediction approaches, the artificial neural network (ANN) based methods have shown promising results when applying for real estate evaluation (Varma et al., 2019; Peter et al., 2020). Their main advantage is the ability to find non-linear relationships between inputs and outputs; therefore, they are suitable for non-linearity prediction for real estate price evaluation and prediction (Ho, Tang & Wong, 2020). Recent studies have claimed machine learning models, including ANNs, to be effective for real estate price prediction tasks (Hamzaoui & Perez, 2011; Morano, Tajani & Torre, 2015; Kang et al., 2020).

However, there have been little effort in modifying these models or adjusting their hyperparameter values for the real state price prediction. For example, Abidoye et al. (2019) used standard autoregressive integrated moving average (ARIMA), artificial neural network (ANN) and support vector machine (SVM) models to generate out-of-sample predictions of property prices, and evaluated them using property price index (PPI). Bin et al. (2019) focused on combining house features and street map image features to perform multi-modal feature fusion using the attention-based neural network, while the evaluation of house price was done using classical boosted regression trees. Ho, Tang & Wong (2020) also used typical machine learning algorithms such as support vector machine (SVM), random forest (RF) and gradient boosting machine (GBM) for evaluation of property price, but did not analyze their hyperparameter optimization. Kim, Kwon & Choi (2020) did consider considered the combinations of the hyper-parameters for a long short-term memory (LSTM) model used to construct a housing price prediction model but they did not use any specific methodology and the applied procedure was ad hoc. The problem, however, was not addressed by Lee & Park (2020), who used the Bayesian neural network as a tool to measure the uncertainty in property valuation, and Liu et al. (2020), who predicted the property price using pre-trained CNN model based on neighboring data samples. Liu (2017) used the bacterial chemotaxis particle swarm algorithm to optimize the initial weights and thresholds of the backpropagation neural network for commercial real estate price evaluation, however, they did not explore the hyperparameters of the network model. Milunovich (2020) focused on the combination of forecast from several machine learning algorithms such as kernel ridge regressions, and deep learning neural networks to predict Australian house prices, but they did not consider optimizing the individual algorithms and models. Štubňová et al. (2020), again, explored only the training of ANNs using different learning algorithms such as Levenberg-Marquart, Bayesian Regularization, and Scaled Conjugate Gradient, but did not consider the optimization of model architecture at the hyper level for residential property price estimation. Similarly, Zhao, Chetty & Tran (2019) used a hybrid model consisting of a pre-trained CNNs model, a MLP model for tabular dataset/numeric features, and another CNNs model to extract visual features from property images, while the XGBoost component performed regression to predict real estate price. However, they did not do any ablation study to motivate the selection of the architecture or its individual components. Zhou (2020) also adopted a standard three-layer feedforward BP neural network, and selected the number of neurons in hidden layer based on the results of pre-experiments with no additional details given on how these were performed.

Despite the robustness of ANN models used in previous studies, the performance of machine learning models is greatly influenced by the selection of hyper-parameter values (Kim, Kwon & Choi, 2020). However, the existing work on real estate (housing) price evaluation or prediction usually applies off-the-shelf machine learning or deep learning methods without considering the optimization of their parameters, or any setting of the parameters is ad hoc. Therefore, the optimization of hyperparameters of deep learning models for real estate price prediction remains a knowledge gap, which the current study is aiming to bridge.

Different numbers of variables, sample sizes, training-testing ratios, and model architectures have been used in various studies. The 80:20 to 90:10 training-testing ratio is the most popular training-testing split. The number of input variables ranges from 6 to 40, which allows us to assume that the optimal feature set is not discovered. The number of variables highly depends on the completeness of data. The model architecture plays an important role in any ANN design: in different approaches, different architectures have been investigated such as 8-13-1 (Morano, Tajani & Torre, 2015), 40-10-1 (Ahmed, Rahman & Sabirah, 2014), and 6-6-1 (Hamzaoui & Perez, 2011) (where a-b-c represents numbers of inputs, hidden layers, and outputs, respectively). The more numbers of hidden layers the ANN architecture has, the more complex it is. High numbers of hidden layers in the previous works indicate the complexity of the solution. The summary of these important studies can be found in Table 1.

**Table 1:** The summary of related research works using ANN for the real estate price prediction. The results cannot be directly comparable due to the different datasets used.

Authors	City/Country	Dataset size/features	Train: test ratio	ANN Model	Accuracy
Mora-Esperanza (2004)	Spain	100/12	85:15	12-7-1	n/a
Mimis, Rovolis & Stamou (2013)	Athens (Greece)	3,150/9	60:20:20	9-5-1	0.86 (R^2^)
Lam, Yu & Lam (2008)	Hong Kong	4,143/29	80:20	30 models	0.78 (R^2^)
Núñez-Tabales, Caridad & Rey (2013)	Spain	10,124/6	80:20	6-6-1	0.86 (R^2^)
Ahmed, Rahman & Sabirah (2014)	Bangladesh	100/40	70:30	40-10-1	0.92 (R^2^)
Morano, Tajani & Torre (2015)	Italy	90/7	80:20	8-13-1	0.99 (R^2^)
Chiarazzo et al. (2014)	Taranto (Italy)	193/42	70:15:15	20-20-1	0.819 (R^2^)
Bin et al. (2020)	Philadelphia (USA)	Places365 database	90:10	VGG16	0.823 (R^2^)
Xu & Gade (2017)	South Boston (USA)	n/a	n/a	4 layers	96.5% accuracy within 20% price range
Abidoye et al. (2019)	Hong Kong	n/a	90:10	3 layers	0.92 (R^2^)
Sun (2019)	n/a	n/a	n/a	3 layers	3.552 (MAE)

The dataset size is an important factor; however, completeness and representativeness are even more important: the more representative instances it contains, the more robust models can be created. As demonstrated in Morano, Tajani & Torre (2015), ANNs can achieve relatively good results even with small datasets. Despite the diversity of the ANN architectures (investigated in similar approaches), it is impossible to identify the best architecture, because architectures were evaluated under very different experimental conditions (in terms of datasets, inputs, etc.) (Zhang & Zhu, 2018; Abiodun et al., 2018).

Moreover, none of the previous studies paid enough attention to the investigation of the hyper-parameter values. Selecting different hyper-parameter values can significantly boost or degrade the overall performance of the ANN even if the architecture remains stable (Feurer & Hutter, 2019). Besides, the number of different hyper-parameter value combinations is very large. Seeking for the optimal ANN hyper-parameter values (optimization function, learning rate, batch size, dropout, validation split, and activation functions) requires additional investigation. Due to it, we consider the hyper-parameter tuning as the essential task of this research and the main goal of it is to improve the baseline approach (with the initial ANN architecture and initial hyper-parameter values chosen by the human expert according to the theoretical insights) by the significant margin. The examples of methods used for optimizing ANN hyper-parameters include various nature-inspired heuristics such as monarch butterfly optimization (Bacanin et al., 2020), swarm intelligence (Bacanin et al., 2020), Bayesian optimization (Cho et al., 2020), multi-threaded training (Połap et al., 2018), evolutionary optimization (Cui & Bai, 2019), genetic algorithm (Han et al., 2020), harmony search algorithm (Kim, Geem & Han, 2020), simulated annealing (Lima, Ferreira Junior & Oliveira, 2020), Pareto optimization (Plonis et al., 2020), gradient descent optimization of a directed acyclic graph (Zhang et al., 2020) and others.

Here we adopted a multilayer perceptron (MLP) neural network model for real estate price prediction in Helsinki (Finland). We present a methodology for MLP model optimization by adjusting hyper-parameters to achieve better performance. To our best knowledge, similar research has never been performed for the Finnish real estate market, which makes this research even more significant and novel as it can help house owners and real estate companies to automate the price predictions with an intelligent and accurate system. Moreover, the insights of this research are valuable with similar datasets and predicting the real estate prices in general.

## Methodology

### Outline

The baseline ANN model was constructed based on the expert knowledge (considering the best practices and recommendations in the related studies) and was used as the baseline (a starting point) to which optimized ANN models were compared and evaluated. Then we applied hyper-parameter optimization on the ANN model seeking to find their values having the best impact on the prediction results. Here we have investigated the following parameters: different deep neural network (DNN) architectures concerning the number of layers (deeper or shallower), optimization functions, loss functions, batch sizes, learning rates, dropouts, and validation splits. Different options of the hyper-parameter values were investigated on the same dataset and the same time interval to keep experimental conditions as equal as possible and to compare different models. The model optimization process includes automatic hyperparameter optimization via different search algorithms. Optimized models are compared to the baseline model.

### The dataset

The ANN requires the dataset to be prepared in a supervised manner: two subsets of it will be used to train and evaluate the model performance. The dataset for our experiments was acquired and pre-processed to train, validate, and test different models. The data used in our experiments contains real estate sales posts and sold apartments in Helsinki in 2019. The property data was harvested using the web crawler (specifically developed for this research) from several marketplaces and data search services. The property data was combined with the area data using postal code as the key attribute. The area data is collected from Statistics Finland and is grouped by a postal code. The latest available data from 2017–2018 about the postal code areas were used. Each instance in the dataset describes an apartment and the area, where it is located. The area is described with 34 features and property with 9 features. The area data is acquired from Statistics Finland (2020) and grouped by the postal code. 29 variables were selected to differentiate postal code areas from each other. Besides, average distances to local services (such as hospitals, schools, grocery stores, and bus stops) in each area are used as attributes. Apartment data was collected from several sources, mainly from real estate marketplaces and data search service about sold apartments offered by the ministry of the environment and the housing finance and development center of Finland (ARA) called “Asuntojen.hintatiedot.fi” (The Ministry of the Environment and the Housing Finance and Development Centre of Finland, 2020) using a dedicated web crawler developed by the authors. Apartment and area data were combined by using postal code as the key variable between two datasets to form the final instances. Data attributes describing an instance were selected based on the importance, consistency, and format. Some data values were converted from a Boolean or categorical format into a numerical format.

The description of the dataset is provided in Table 2. The description of the dataset is divided into property- and area attributes. The descriptive statistics of the dataset variables under examination is presented in Table 3.

**Table 2:** Description of the dataset attributes.

Property		
Variable	Category	Description
Debt free price	Price	Price of an apartment, dept free price
Living area	Size	Size (m²) of an apartment
Rooms	Size	Number of rooms in an apartment
Living floor	Building	Floor number where an apartment is located
Total floors	Building	Total number of floors in a building
Type	Building	Type of an apartment, numerical value
Year	Building	Building year
Energy class	Building	Energy class of a building, numerical value
Property ownership	Building	Property ownership, own or rental, numerical value
**Area**		
Population	Population structure	Population of the postal code area
Average age	Population structure	Average age of inhabitants
Aged 18 or over	Education level of residents	Amount of over 18-years old, total
With education	Education level of residents	People with at least an upper secondary qualification
Lower level university degree	Education level of residents	University/tertiary-level degree, lower: lower-degree level tertiary education (level 6)
Higher level university degree	Education level of residents	University/tertiary-level degree, higher: higher-degree level tertiary education (level 7) and doctorate degrees or equivalent (level 8)
Median income of inhabitants	Resident disposable income	Median income of inhabitants (€) is obtained by listing inhabitants by the amount of disposable monetary income
The lowest income category	Resident disposable income	Inhabitants earning at most EUR 13 287 per year
The middle-income category	Resident disposable income	Inhabitants earning EUR 13 288 - 31 873 per year
The highest income category	Resident disposable income	Inhabitants earning more than EUR 31 874 per year
Purchasing power of inhabitants	Resident disposable income	Accumulated purchasing power of inhabitants (€) is the accumulated disposable monetary income
Households	Size and stage of life of households	Number of households in total
Occupancy rate	Size and stage of life of households	Occupancy rate (m2) is the average floor area that is derived dividing the total floor area by the number of inhabitants
Owner-occupied dwellings	Size and stage of life of households	Households living in owner-occupied dwellings are households whose tenure status is owner-occupied dwelling
Rented dwellings	Size and stage of life of households	Households with rented dwellings are households whose tenure status is rental, subsidized, interest subsidized rental and right of occupancy dwellings
Median income of households	Disposable monetary income of households	Median income of households (€) is obtained by listing households by the amount of disposable monetary income
The lowest income category	Disposable monetary income of households	Households earning at most EUR 16 979 per year
The middle-income category	Disposable monetary income of households	Households earning EUR 16 980 - 35 297 per year
The highest income category	Disposable monetary income of households	Households earning more than EUR 35 298 per year
Purchasing power of households	Disposable monetary income of households	Accumulated purchasing power of households (€) is the accumulated disposable monetary income
Buildings, total	Buildings and housing	The total number of buildings per area. Free-time residences are not included in this total
Residential buildings	Buildings and housing	Residential buildings is the number of buildings per area that are intended for residential use
Blocks of flats	Buildings and housing	Dwellings in blocks of flats are dwellings in residential blocks. They include buildings with at least three flats of which at least two are located on top of each other
Average floor area	Buildings and housing	Average floor area (m2) is the total floor area of all dwellings divided by their number
Workplaces	Jobs by industry	Number of workplaces is the number of people working (including part-time) in each area
Employed	Main activity of residents	Employed labor force is defined as people aged 18 to 74 who were employed during the last week of the year
Unemployed	Main activity of residents	Unemployed labor force comprises people aged 15 to 64 who were unemployed on the last working day of the year
Students	Main activity of residents	Students are defined as persons who study full-time and are not gainfully employed or unemployed
Pensioners	Main activity of residents	Pensioners are defined as persons who according to the Finnish Centre for Pensions receive a pension or have some other pension income
Distance to a bus stop	Local services	Average distance (m) to the nearest bus stop
Distance to a grocery store	Local services	Average distance (m) to the nearest grocery store
Distance to a doctor or hospital	Local services	Average distance (m) to the nearest doctor or hospital
Distance to a school	Local services	Average distance (m) to the nearest school
Distance to a sports center	Local services	Average distance (m) to the nearest sports center

**Table 3:** Descriptive statistics of the selected dataset attributes.

Variable	Min	Max	Mean	Std. dev.
Debt free price, EUR	100,000	995,000	308,750	152,130
Living area, m^2^	14	199	65.44	28.38
Rooms	1	6	2.56	1.06
Year	1925	2020	1979	28.61

### Analysis of dataset

**Figure 1:**
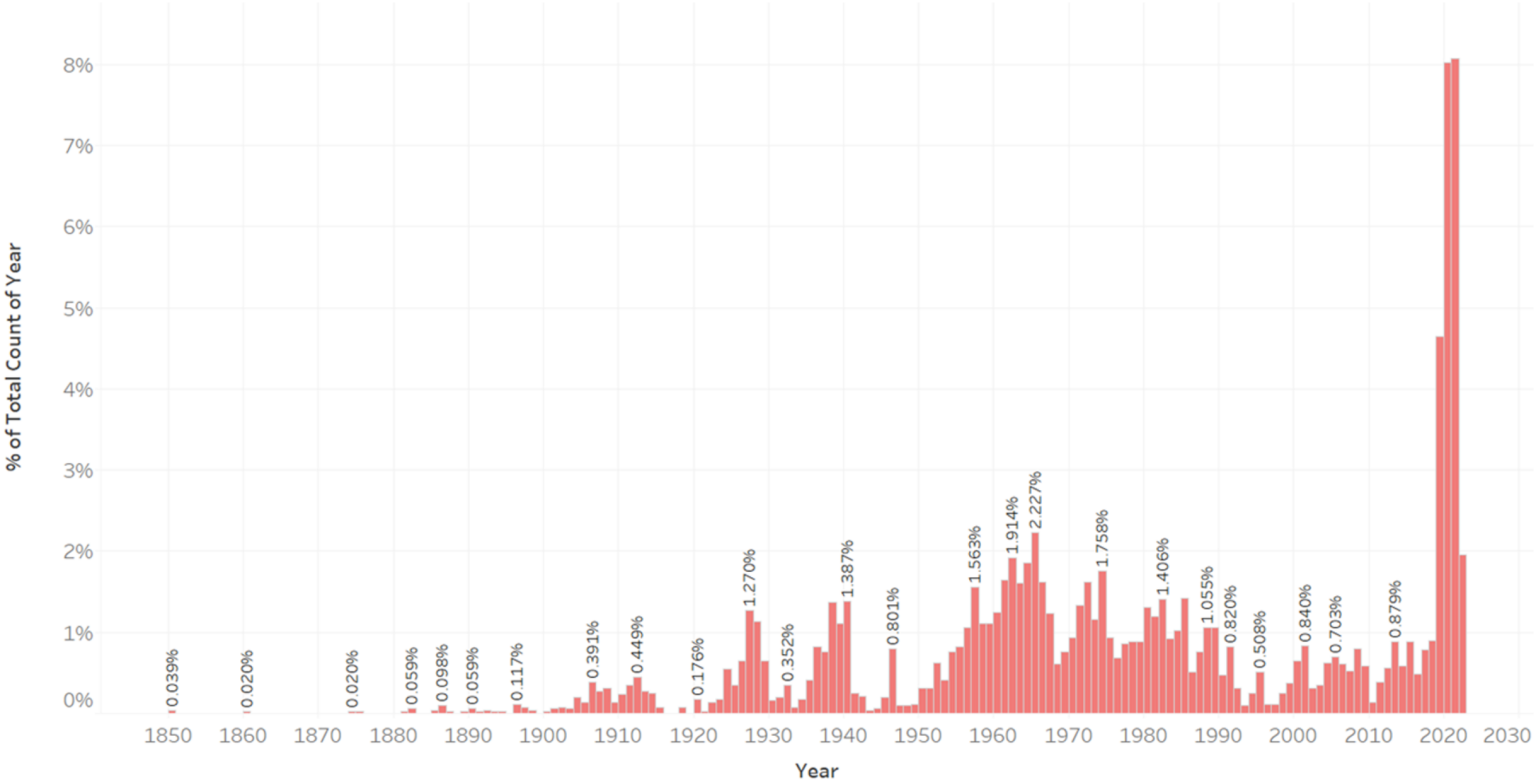
Distribution of apartments according to their construction year.

The construction year is an important factor that introduces non-linearity to our problem. Old apartments can be significantly more expensive than similar, but newer, apartments in the same area. In Helsinki, the range of the construction year is wide, from 1850 to 2020. Only a few of the apartments are built between 1850–1925, 9.4% of the total number of instances. 90.6% of the apartments fall between 1925 and 2020 (Fig. 1).

**Figure 2:**
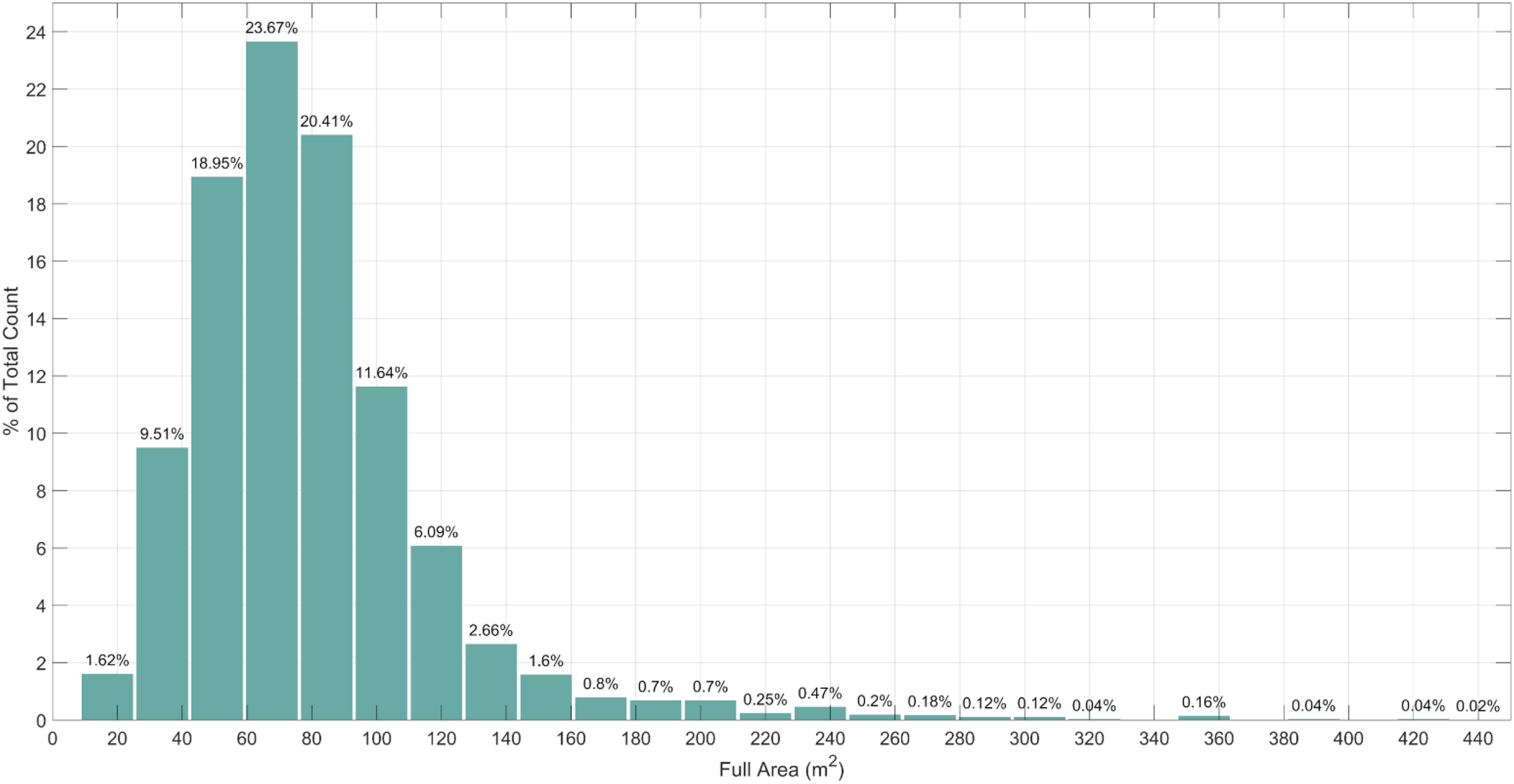
Distribution of apartments according to their size.

Another important factor is the size of an apartment. Outliers and anomalies occur in the size category also. Apartments with a size of 0–10 m^2^ is most likely a mistake made by a real estate agent or web crawler, and are excluded. Lack of data about extremely large apartments, more than 200 m^2^, can decrease the accuracy of a common apartment. Excluding instances that contains previously mentioned values, we end up with 15–200 m^2^ size of apartments, which make 96.74% of the whole dataset (Fig. 2).

**Figure 3:**
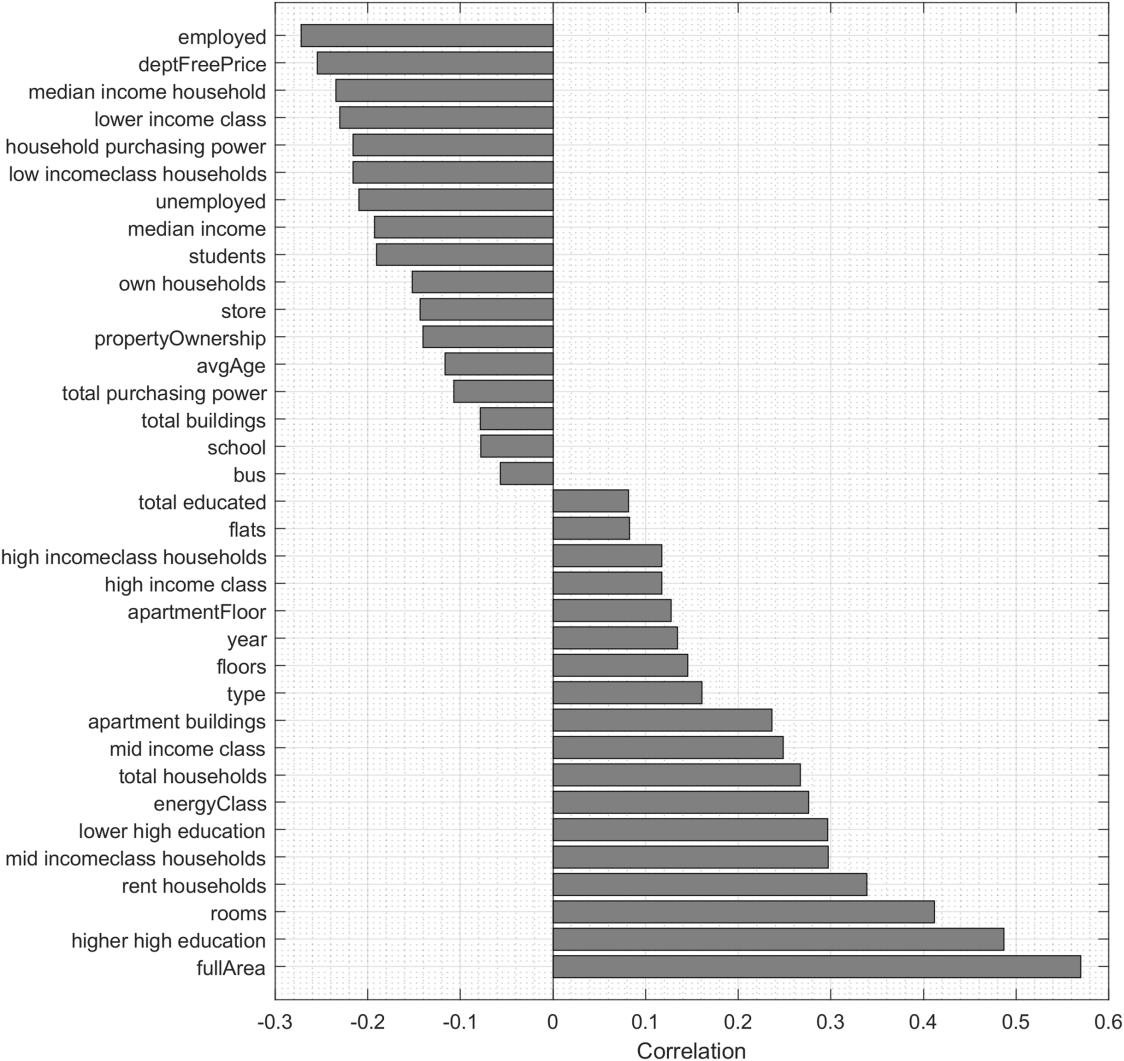
Statistically significant (*p* < 0.001) correlations of features with the apartment price.

To analyse the importance of features for predicting the price of apartments, we use Pearson correlation, neighborhood component analysis (NCA) and regression trees. Pearson correlation allows to analyze the features, which have both positive influence and negative influence on the price of the apartment. Figure 3 shows the correlation values of features with the apartment price, which are statistically significant (*p* < 0.001). The most correlated feature of the dataset is the full area of the apartment (r = 0.5696).

**Figure 4:**
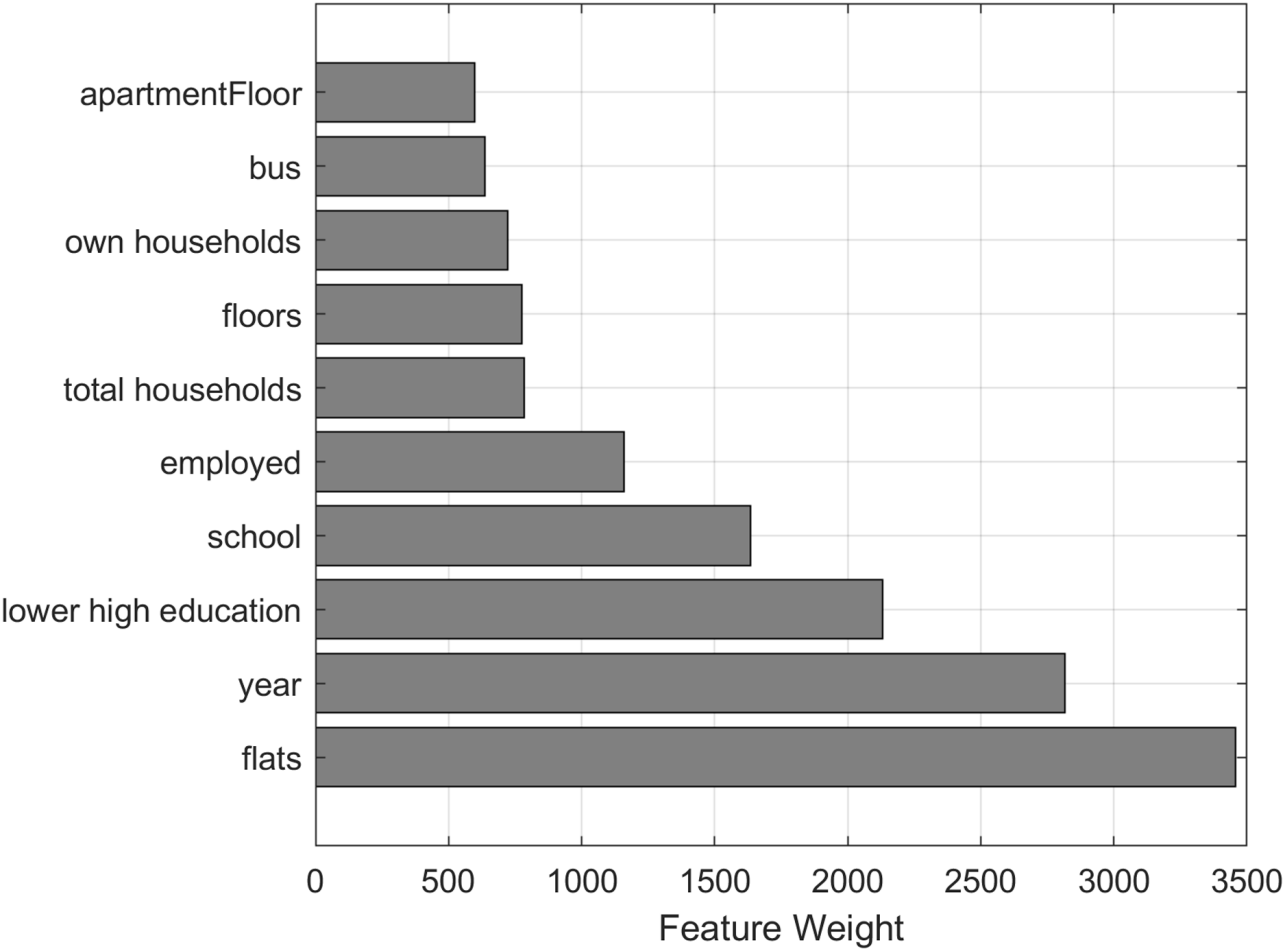
Top 10 property features with the biggest weight values calculated using neighborhood component analysis.

NCA (Goldberger et al., 2005) aims to learn a distance metric in the feature space by finding a linear transform of input features so that average classification performance is maximized in the transformed feature space. The NCA model is used to calculate feature weights using a diagonal adaptation of NCA and then regularizing the feature weights. The top 10 features with the biggest weight value are visualized in Fig. 4, showing that the number of flats in total (“flats”) and the construction year of the apartment building are the most important features for predicting the price of an apartment.

**Figure 5:**
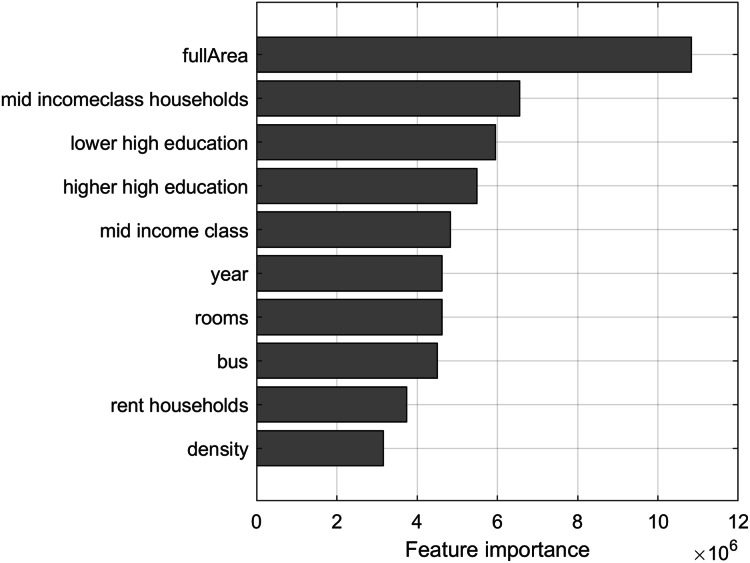
Top 10 most important features of the dataset in terms of predictor importance from regression tree.

Predictor importance (Bi, 2012) is estimated by constructing the regression tree and then summing changes in the mean squared error (MSE) due to splits on every predictor and dividing the sum by the number of branch nodes. At each tree node, MSE is calculated as node error weighted by the node probability. Predictor importance associated with this split is computed as the difference between MSE for the parent node and the total MSE for the two children nodes. The top 10 most important features of the dataset in terms of predictor importance are visualized in Fig. 5, showing that the full area of the apartment is the most important feature for predicting the price of an apartment.

### Data preprocessing

Data pre-processing stage contained such steps as detection and removal of outliers and incorrect (empty) instances and, finally, standardization of the data. The purpose of the data cleanup is to convert raw data into a good quality dataset which is essential for the ANN model to perform accurately. The outliers and false instances were eliminated to assure the best possible conditions to train an accurate and robust model. Outliers (such as extremely expensive or large apartments that are sold rarely) can negatively affect the overall performance, therefore the outliers were excluded. False instances (which contain empty or null values) were errors introduced by the web crawler. The final dataset was formed after pre-processing. After removing mistakes and outliers, the final dataset contains 4,041 instances from 67 postal code areas.

Since the format and the scale of the data varied, it required standardization. Standardization helped to map values into a similar range. Here we used a feature range between zero and one, where one represents the highest value and zero the lowest, which is derived (Eq. (1)) as follows:

(1) $$Xsc=\;\frac{X-Xmin}{Xmax-Xmin}$$

where X is the attribute value, Xmin is the minimum value for the attribute in the dataset and Xmax is the maximum value for the attribute in the dataset.

Attributes are then divided into two groups, i.e., prediction- and target attributes. Prediction attributes are used as an input for the ANN and the target attribute is the determined value. In total, 43 attributes describe each instance (42 of which are used as the input/source and 1 attribute as the target). Finally, preprocessed data was shuffled and divided into training and testing subsets with the 80:20 ratio (as it is typically done in similar research works (Lam, Yu & Lam, 2008; Núñez-Tabales, Caridad & Rey, 2013)).

## Artificial neural network

The MLP architecture contains an input layer, one or more hidden layers and an output layer. All connections are pointing towards the output node which means MLP is a feedforward neural network. Layers are fully connected to each node in the next layer. Every connection has a weight assigned to it and a weighted sum is calculated and passed through a non-linear function. The non-linear function is called an activation function and introduces non-linearity to the solution. The non-linear activation function is used in every other layer than the input and output layer. Common examples of the activation functions are single-pole sigmoid, hyperbolic tangent (tanh), Exponential linear unit (eLU), Scaled exponential linear unit (seLU), and Rectified linear unit (reLU). Currently, the reLU (Eq. (2)) activation function is considered as the best practice (Wang et al., 2020).

(2) f(α,x)=max(0,x)

Activation functions are non-linear functions that belong to a group of hyper-parameters that can be adjusted for better performance. Other examples of hyper-parameters are optimization functions, learning rates, batch sizes, validation splits, loss functions, and model architectures. The model architecture consists of the number of hidden layers and the number of nodes in each hidden layer. Each hyperparameter has its purpose in the model and fine-tuning these values can make a significant difference in the models and their results. Hyper-parameter optimization is performed in this research by using automatized search algorithms to find the most accurate model for solving the real estate price prediction problem.

## Experiments and results

### Settings

For implementation, we used Python version 3.7.4. Several third-party Python libraries are used, such as are Numpy scientific computing and Pandas for data structures and analysis. Keras is used as a high-level neural network API. Node.js is used to create a backend server for the application that performed crawling of data from the Asuntojen.hintatiedot.fi website. Tableau was used for data visualization and analytics.

### Evaluation metrics

We have experimented with different ANNs architectures and hyper-parameters by training and testing the obtained models on our dataset. Models were evaluated with several metrics, i.e., Mean Squared Error (MSE) on the test set, MSE on the training set, the difference between MSEs, validation loss, and training loss. For the best-determined model, the sensitivity analysis was also performed by using Mean Absolute Error (MAE), R-squared (R^2^), Root Mean Squared Error (RMSE), and Relative Mean Error (RME). R2 (Eq. (3)) measures a fit for linear regression models. It describes the percentage of variance and measures the relationship between prediction and targeted price on a scale of 0–100%. RMSE (Eq. (4)) is the root of the average of squared differences between predictions and target prices. MAE (Eq. (5)) is the average error magnitude of prediction and target prices. RME (Eq. (6)) is the absolute error between predicted and targeted prices in percentages. MSE (Eq. (7)) is the average of the squares of the errors, i.e., the average squared difference between the estimated values and the actual value. These metrics are commonly used in real estate property evaluation studies (Nejad, Lu & Behbood, 2017; Xue et al., 2020).

(3) $$R^{2}=1-\frac{\sum_{i}{\left(y_{i}-{\hat{y}}_{i}\right)}^{2}}{\sum_{i}{\left(y_{i}-{\bar{y}}_{i}\right)}^{2}}$$

(4) $$RMSE=\sqrt{\sum_{i=1}^{n}\frac{{\left({\hat{y}}_{i}-y_{i}\right)}^{2}}{n}}$$

(5) $$MAE=\frac{1}{n}\overset{n}{\underset{i=1}{\sum }}\left|y_{i}-{\hat{y}}_{i}\right|$$

(6) $$RME=\frac{1}{n}\overset{n}{\underset{i=1}{\sum }}\frac{\left|y_{i}-{\hat{y}}_{i}\right|}{\left|{\hat{y}}_{i}\right|}*100\%,$$

(7) $$MSE=\frac{1}{n}\overset{n}{\underset{i-1}{\sum }}{\left(y_{i}-{\hat{y}}_{i}\right)}^{2}\;$$

where $y_{i}$ is the forecasted price by the model and ${\hat{y}}_{i}$ the actual, targeted, price of the i-th real estate and the number of properties is *n*.

### Initial model evaluation

The initial (or a baseline) model architecture is selected based on expert knowledge, considering the best practices in the previous studies. In many similar studies, the ANN model architecture typically contains 6 to 15 hidden layers. A similar model architecture can still be used in our research as an initial step to set the starting point before the model optimization. The numbers of hidden layers and neurons in these layers are determined considering the complexity of our solving real estate price prediction problem. Assumptions about the complexity of our solving problem are made considering the previous research (see Table 1). It resulted in choosing the larger numbers of hidden layers and neurons in the initial ANN architecture. The number of nodes in each hidden layer was set to 128. Every layer, except for the input layer, had the activation function (having a large effect on the performance). Following the best practice, we use the reLU function, which has the benefits of sparsity and good behavior when dealing with the vanishing gradients problem. Moreover, reLU is more computationally efficient to compute than Sigmoid functions and it has better convergence performance (Krizhevsky, Sutskever & Hinton, 2012).

Other hyper-parameters are the batch size, optimization algorithm and learning rate, loss function, dropout, and the number of epochs. In our experiments, the initial hyper-parameters were set to the following values: the batch size = 128, Adam (Kingma & Ba, 2014) as the optimization function with the learning rate of 0.001, MSE as a loss function, and no dropout. The early stopping function was used after a certain number of epochs to determine the training process if the model demonstrated no improvement in the performance.

The initial model performance is presented in Table 4. The performance was evaluated using the following procedure. The model was trained and evaluated five times (to avoid abnormalities due to random weight initialization): the obtained results were averaged. Training results show that the model is not underfitting or overfitting, because there is no large difference between MSE values on training and testing datasets. These results were later compared to the optimized model to measure the progress of the optimized models.

**Table 4:** Prediction performance of an initial (baseline) ANN model.

No.	Training results					Testing results				
	MSE	MSE Train	MSE Difference	Val loss	Loss	R^2^	RMSE	RME		MAE
1	0.0027	0.0024	0.0003	0.00895	0.0026	0.90	46,517.8	10.9%		31,076.6
2	0.0026	0.0020	0.0006	0.00896	0.0012	0.90	46,054.24	9.89%		29,642.0
3	0.0028	0.0025	0.0002	0.00774	0.0023	0.90	47,202.2	11.5%		32,093.7
4	0.0029	0.0020	0.0009	0.01021	0.0012	0.90	48,106.7	10.3%		30,562.5
5	0.0027	0.0025	0.0002	0.00909	0.0020	0.90	46,197.5	11.3%		31,468.3
AVG	0.0027	0.0023	0.0004	0.00899	0.0019	0.90	46,815.7	10.8%		30,968.6

### Evaluation of optimized model

Optimization of the model can be performed in two ways: manually (by analyzing training results and then tuning hyper-parameters towards the more accurate model) or automatically (via the search and optimization algorithms). Both approaches use the trial-and-error method, and both are considered correct if they lead to the creation of the optimal model. However, manual tuning is time-consuming. Besides, human experts tend to bind to more probable hyper-parameter values that can cause a risk (especially in non-typical cases) that the optimal set of hyper-parameters will not be found.

Due to these reasons in our research, we have used the automatic Weights & Biases developer tool (Weights & Biases, 2020). It iterates through the defined value ranges and categories, using the determined search algorithm, which is Bayesian optimization. Bayesian hyperparameter tuning builds a probabilistic model for the objective function to be optimized in order to train the deep learning model (see Table 5). Bayesian optimization attempts to collect measurements that reveal information about the objective function and the position of the optimum by iteratively testing a promising hyperparameter structure based on the current model, and then modifying it. Exploration (hyperparameters with which the effect is most uncertain) and exploitation was attempted to match (hyperparameters expected close to the optimum). The algorithm optimizes the following hyper-parameters: batch size, learning rate, optimization algorithm, activation function, validation split, dropout, and model architecture. The model architecture is divided into several layers and many nodes in each layer separately. The starting value ranges and categories of each iteration are summarized in Table 6. The range of values is justified by the analysis of previous works on real estate price prediction, which is presented in Table 1.

**Table 5:** Hyperparameter optimization algorithm.

Build a probability model Dof the objective function u() Find the hyperparameters that perform best on the probability model $x_{k}=\underset{X}{arg\;max}u\left(X|D_{1:k-1}\right)$ Apply these hyperparameters to the objective function and get the performance $y_{k}=f\left(x_{k}\right)$ Update the probability model incorporating the new results $D_{1:k}=\left\{D_{1:k-1},\left(x_{k},y_{k}\right)\right\}$ Repeat steps 2–4 until max iterations or max computation time is reached	

**Table 6:** Hyperparameter value ranges and categories in each optimization iteration.

No.	Dropout	Batch size	Validation split	Learning rate	Number of layers	Optimizer	Activation function	Number of nodes	Search
1	0–0.5	10–1,000	0.05–0.2	0.0007–0.0011	1–10	Adam, SGD, RMSProp, NAdam	reLU, elu, selu, sigmoid, tanh	1–3: 64–1,024 4–6: 64–512 7–10: 64–256	Random
2	0–0.2	10–1,000	0.08–0.19	0.0008-0.0011	1–10	Adam	reLu	All: 50–1,000	Bayes
3	0–0.1	10–1,000	0.08–0.12	0.0009–0.0011	6–10	Adam	reLu	1: 600–1,000 2: 50–300 3: 500–1,000 4: 200–800 5: 700–1,000 6: 600–1,000 7: 200–800 8: 50–600 9:300–900	Bayes
4	0–0.05	300–700	0.08–0.1	0.001–0.002	6	Adam	reLu	1: 700–900 2: 100–300 3: 600–900 4: 300–600 5: 900–1,000 6: 700–1,000	Bayes
5	0.3	600	0.085	0.0014	6	Adam	reLu	1: 500–750 2: 250–500 3: 500–750 4: 400–700 5: 950–1,000 6: 800–1,000	Bayes

The purpose of the hyper-parameter optimization process is to get a wide variety of results and to seek correlations between results and hyper-parameter value combinations. The random search is used in the first iteration because the search algorithm must not form any bias towards certain values at an early point. Other search algorithms are used in further iterations. The best 10% of the runs are analyzed to find correlations between results and hyper-parameters. The Bayesian search algorithm used the MSE metric to find the best performing hyper-parameter values. MSE was calculated from the error between prediction and target prices on the testing set. The MSE value calculated from the testing set represents how the NN can predict prices of the unseen data. Other metrics, such as validation loss, training loss, the difference between losses and MSEs are used to narrow the search value ranges for further iterations.

The first iteration had the widest value ranges of values for each hyper-parameter, and it used the random search. The random search is used so that the search algorithm does not create any bias towards certain value groups. The random distribution over hyper-parameters values gives enough variety in the results, that the search can be narrowed afterward. Every run with MSE lower than 0.0045 was saved. The critical values represent the 10th percentile of the values obtained from all runs, that is the selected set of results contained top 10% of results, which were better than the remaining 90% of results. The best 10% of the runs were analyzed, so the final sample size contained 27 runs. A deeper analysis reveals that reLU as the activation function and Adam as the optimizer appeared in the majority of the best runs. The majority of the analyzed runs containing similar hyper-parameter values are used to find correlations between results and hyperparameter sets. Other hyper-parameters did not have a similar, obvious, correlation with the results. Thus, before continuing the search for the rest best hyper-parameter values in the further iterations, the activation function and the optimizer were set to reLU and Adam, respectively.

The second iteration achieved even better results compared to the first iteration. 312 runs were saved from 343 runs in total. Each saved run had MSE between 0.0022 and 0.0040. The Bayes search algorithm was used to optimize the hyper-parameters. The best 10% of the runs were analyzed in detail, therefore the sample size of this iteration resulted in 32 runs. From six to ten hidden layers were used in 75% of these runs and were considered as a new range of values for the next iteration. Surprisingly, the number of nodes did not fall into the same value range in each layer. Layer 1 contained 600 to 1,000 nodes, but layer 2 had 50 to 300 nodes. The same phenomenon was observed in the first and last layers. The hyper-parameter values for the best model architecture could still be narrowed down in the next iterations since no clear correlation was noticed.

The third iteration produced consistent results and therefore was fast and efficient to compute. Models were only saved if MSE value was lower than 0.0031. Models trained with these hyper-parameter values do not perform significantly better compared to previously created, but the results are more consistent. 209 of 301 runs had MSE between 0.0021 and 0.0031. The best 10% (or the 21 runs of) all were taken for further analysis. This analysis revealed six hidden layers to be the dominant value for the best architecture because 48% of all analyzed runs used it. The correlations between the best results and other hyper-parameter values were determined as follows. The range for the validation split was decreased to 8–10%; the dropout and the batch size got into the range of 0–0.05 and 300–700, respectively. The analysis shows that 66% of the runs had a validation split between 8–10%, 81% had a dropout between 0–0.05, and 62% had a batch size between 300–700. The range of nodes in the hidden layers could be decreased by a small margin. All mentioned hyper-parameter values were set and considered as new value ranges for the next iterations.

The fourth iteration produced the best performance and improved the results by 12.3% compared to the best run from previous iterations. The best run was better than any other run on the same iteration by a decent margin. The hyper-parameters on the run were batch size 550, dropout 0.005, learning rate 0.0012, and validation split 8%. The model architecture contained six hidden layers with the following numbers of nodes: 900, 150, 700, 550, 950, and 950. MSE on the testing and training sets gave 0.001877 and 0.001538, respectively; the difference between MSEs was 0.00034. The performance differences in testing and training sets show that the model is neither overfitting nor underfitting and can produce good results with unseen data. Finally, the fifth iteration was performed to fine-tune the model architecture, but no improvement was found after 152 runs. Therefore, the best model from the fourth iteration can be considered as the optimized model.

**Figure 6:**
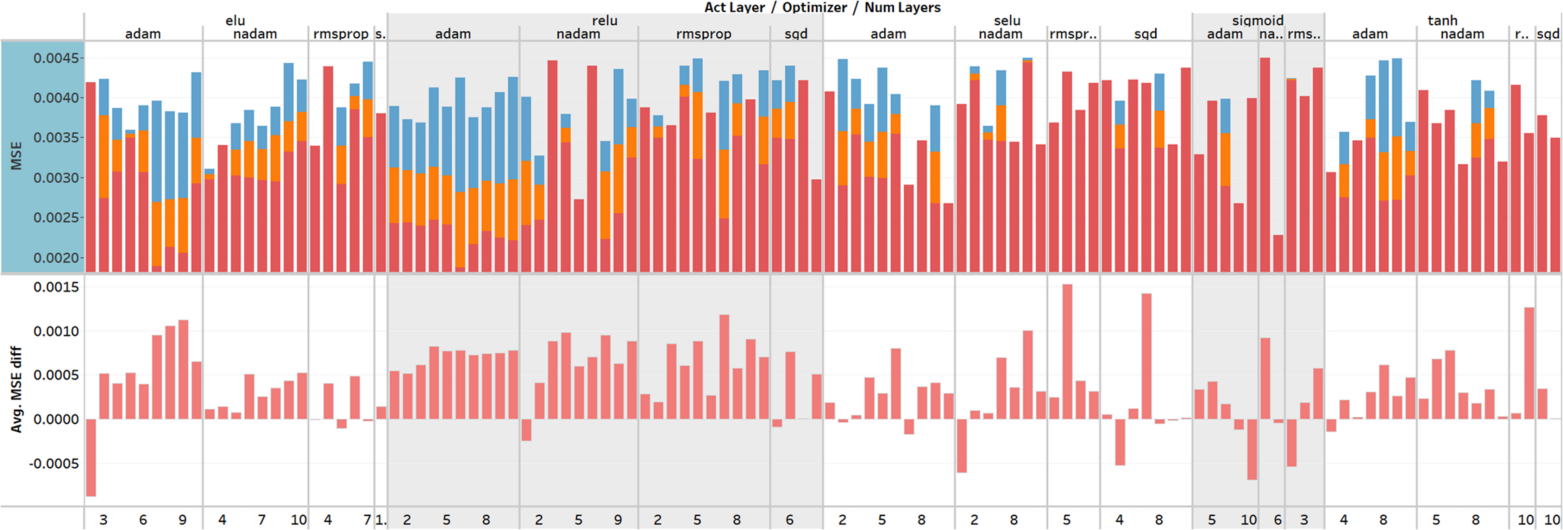
Overview of the hyperparameter optimization. The *X*-axis represents the activation function, optimization function and number of layers, and *Y*-axis represents MSE values and the difference between MSE on the training and testing set. Plotted MSE values are minimum, average, and maximum from all training sessions. The upper section describes the performance of the model using the MSE metric. The lower section shows the difference between MSE values which describes the over- or under-fitting of the model.

To summarizing, in total 2003 runs were performed with different hyper-parameter value combinations and 1,514 runs were saved, where the MSE was equal to or lower than 0.0045. The worst run, which was saved had 0.004495 MSE and the best had 0.001877. The average MSE was 0.002964. The most optimal model was formed with the hyper-parameter values as follows: reLU as the activation function, Adam as the optimization algorithm, batch size 550, dropout 0.005, learning rate 0.0012, and validation split 8%. The best model architecture contains the single input layer with 42 nodes, six hidden layers with the following number of nodes 900, 150, 700, 550, 950, 950, and the single output node. Finally, an overview of all performed training sessions can be seen in Fig. 6.

The optimized model was evaluated and compared to the initial one with nine evaluation metrics. These metrics were separated into two categories: for training and testing evaluation. Table 7 represents the results of both models and their differences. The best model outperformed the first initial model on every metric. The initial model was created based on the expert knowledge and recommendations in the previous research works. It allows us to conclude that despite how good the DNN architecture and the set of hyper-parameters performs in similar tasks, recommendations cannot be blindly followed.

**Table 7:** Results for initial and optimized models. Baseline model architecture is selected based on expert knowledge, considering the best practices in the previous studies. Optimized model is developed using hyper-parameter optimization.

Model	Training results						Testing results			
	MSE	MSE Train	MSE Difference	Val loss	Loss		R^2^	RMSE	RME	MAE
Baseline	0.0027	0.0023	0.0004	0.00899		0.0019	0.90	46,815.7	10.8%	30,968.6
Optimized	0.0018	0.0015	0.0003	0.00669		0.0011	0.95	33,232.2	8.3%	23,320.9
Difference	−0.0009	−0.0008	−0.0001	−0.0023		−0.008	0.05	−13,583.5	−2.5%	−7647.7
Improvement	33.3%	34.8%	25.0%	25.6%		42.1%	5.56%	29.0%	23.2%	24.7%

Training results are mostly used to evaluate the training model, but the focus should be on the sensitivity analysis of the testing results. The best model improved the RME value, calculated from the actual differences between predicted and targeted price, by 23.2% and decreased it to 8.3%. This is a large improvement because every error percent impacts thousands of euros in the final price, and MAE was also improved by 24.7% and decreased to 23320.9 €. The R^2^ metric was improved by 5.56% to 0.95. The sensitivity analysis measures how well the model observes the targeted outcome. All the metrics were improved by the significant (*p* < 0.05) margin, which allows us to conclude that improvement is significant compared to the initial model.

**Figure 7:**
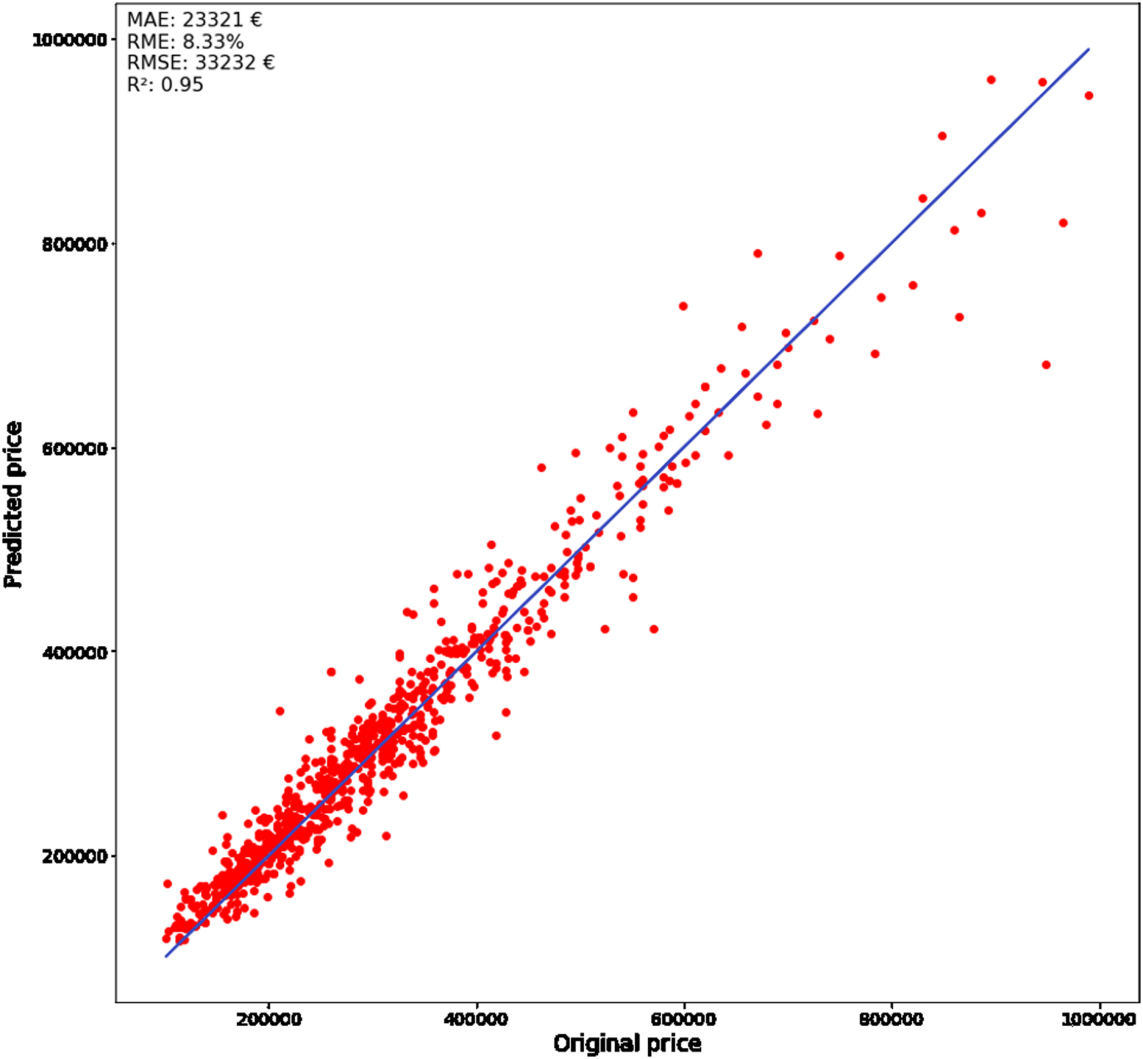
Real (solid line) and predicted (dots) prices.

Figure 7 represents the error between predicted and original price, where the solid line and the markers determine the real price and the predicted price, respectively. The lower prices are predicted more accurately compared to the higher. The correlation between the amount of the data and the accuracy can be the more instances the certain property type has, the lower the error rate is achieved. The testing dataset was divided into different categories which were further analyzed to get a better understanding of this. Overall, the metrics show good performance on the whole dataset, where 95% of the predicted prices are on the regression line (with RME and MAE equal to 8.3% and 23,320.9 €, respective) Despite the higher error rates are with more expensive apartments, the obtained results can still be considered as satisfactorily. The sensitivity analysis is performed on a divided dataset to get an understanding of the accuracy of more common cases, where the lack of data is not affecting the results. The dataset was divided by the number of rooms because it is a good measure related to differences in apartment prices and their sizes. Afterward, the sensitivity analysis was performed on each of the obtained subsets.

**Figure 8:**
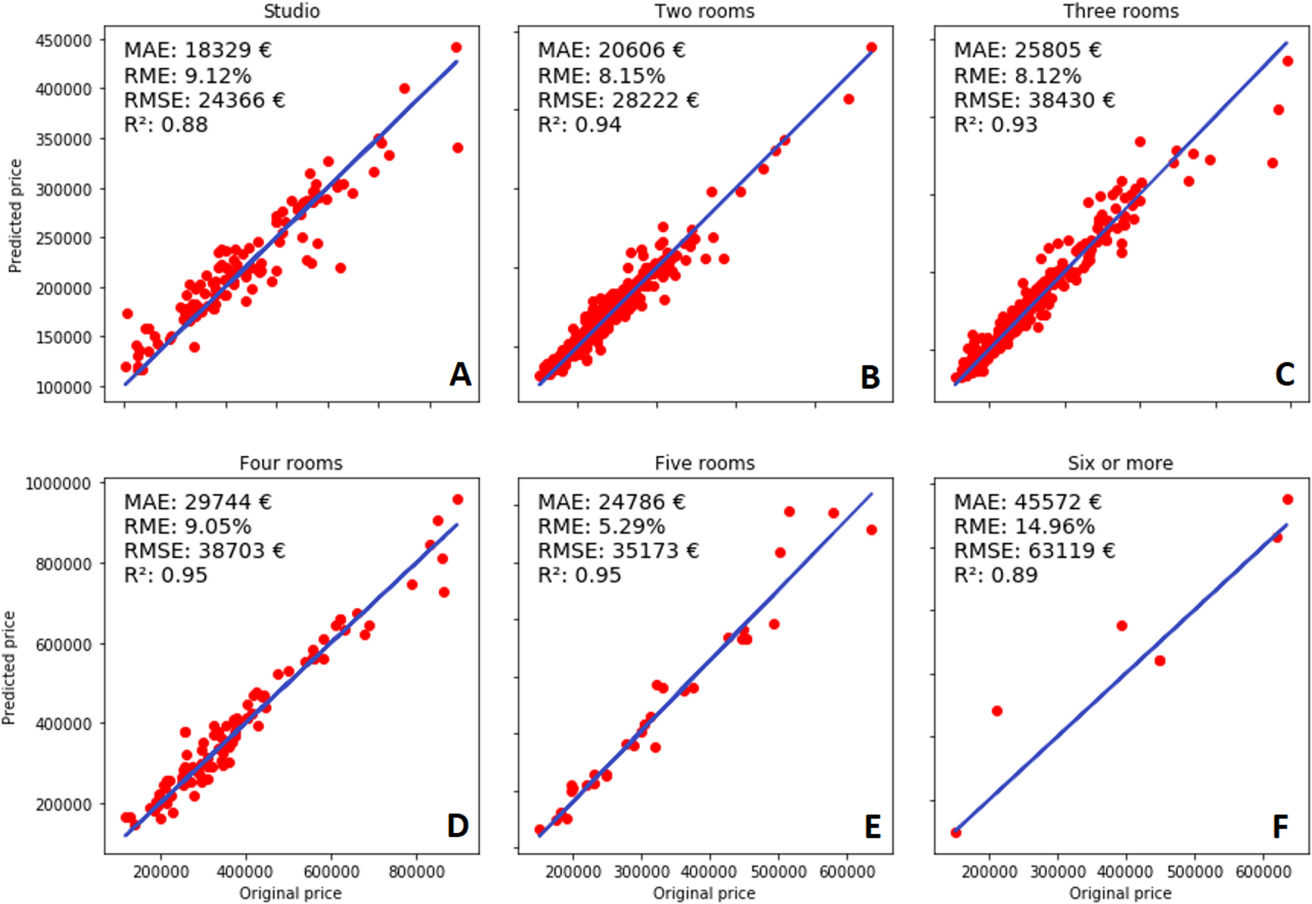
Number of rooms and performance of the model: (A) Studio, (B) two rooms, (C) three rooms, (D) four rooms, (E) five rooms, (F) six or more rooms.

The analysis of the results based on the number of rooms (presented in Fig. 8) reveals interesting information about the model. The obvious fact is that the number of instances decreases when the number of rooms increases, and it is the most probable reason causing the previously described issues. RME is the highest when the apartment has six or more rooms (compared to any other category containing fewer rooms in the apartment). However, it still has a better R^2^ value when compared to the studio apartments, which are even more expensive. All metrics except R^2^ measure the difference between the actual prices, which can be misleading, because of the very large price range. Therefore, R^2^ is used to compare the results between different room prices. For each category, the calculated R^2^ value shows that the model can predict the observed instances quite well. The best performance was achieved in the categories containing four-five rooms, and two- and three-rooms; whereas studio and six-room apartments are predicted slightly worse. As it was mentioned previously, the dataset contained fewer instances for larger apartments, therefore the related category was predicted worse. Surprisingly, the same reasoning is not valid for studio apartments.

The dataset contained almost the same number of instances as the other room categories (even more than four- five-room apartments), but predictions are still worse compared to those categories. Most of the predicted prices were slightly higher than the targeted values. The random division into the training and testing datasets could also have a negative effect on the results. The training set happened to contain more expensive apartments than the testing set, which would create a bias towards higher prices. The phenomenon of the imbalanced dataset does not occur in the other categories.

The price of overall, two-, three-, four-, and five-room apartments were predicted well. The predicted prices are on 93–95% in the regression line and the difference between MAE and RMSE is not significant. Here the statistical significance was evaluated using 95% confidence intervals (CI). RME is between 5.29–9.05%, and, surprisingly, the best results were achieved with the five-room apartments. Again, this can be a cause of a poorly divided dataset where the training dataset had a lot of instances describing these types of apartments. Two-, and three-room apartments are the most frequent in our dataset, therefore the DNN model has enough material to learn how to predict their prices correctly. Besides, the model was also capable to evaluate rarer sold apartments, which shows its ability for generalization.

## Conclusions

In this paper, we presented the methodology and results of optimizing the MLP model aimed at predicting the real estate prices in Helsinki, Finland. Optimization of model hyper-parameters improved the performance by a good margin (the R^2^ value improved by 0.05 and the RME value improved by 2.5%), and therefore can be considered as an important step in developing real-estate prediction applications. However, the ANN approach has its downsides and therefore receives criticism. The ANN’s lack of explainability as relationships between inputs and outputs cannot be directly perceived and explained; besides humans cannot directly intervene in these relationships. However, producing sustainable property price evaluations without human intervention can be considered as a benefit. The ANN can produce more accurate, flexible, and generic results, if enough data is available compared to the other approaches, therefore, they can be considered as a good solution for the price prediction problem. The result analysis shows that model optimization process improved the performance significantly on each metric. Training results shows no over- or underfitting and sensitivity analysis describes good performance on the testing set. Analysis shows that results can be improved by focusing on model optimization and hyperparameter tuning. The research has shown that real estate prices can be predicted in Helsinki, Finland, using deep neural network approaches and deep learning can be used in similar regression tasks for forecasting non-linear relationships between inputs and outputs.

In future research, using more data and extending the hyperparameter optimization process to other types of neural networks could lead to finding a more robust and accurate real estate price evaluation model.

## Supplemental Information

10.7717/peerj-cs.444/supp-1 Supplemental Information 1 Dataset of real-estate in Helsinki.

10.7717/peerj-cs.444/supp-2 Supplemental Information 2 Python code for neural network training.

## Additional Information and Declarations

### Data Availability

The following information was supplied regarding data availability: Data and code are available in the Supplemental Files.

## References

[ref-1] Abidoye RB, Chan APC, Abidohye FA, Oshodi OS (2019). Predicting property price index using artificial intelligence techniques: evidence from hong kong. International Journal of Housing Markets and Analysis.

[ref-2] Abidoye RB, Chan APC, Abidoye FA, Oshodi OS (2019). Predicting property price index using artificial intelligence techniques: evidence from hong kong. International Journal of Housing Markets and Analysis.

[ref-3] Abiodun OI, Jantan A, Omolara AE, Dada KV, Mohamed NA, Arshad H (2018). State-of-the-art in artificial neural network applications: a survey. Heliyon.

[ref-4] Ahmed S, Rahman M, Sabirah I (2014). House rent estimation in Dhaka City by multi layer perceptions neural network. International Journal of u- and e-Service, Science and Technology.

[ref-5] Bacanin N, Bezdan T, Tuba E, Strumberger I, Tuba M (2020). Monarch butterfly optimization based convolutional neural network design. Mathematics.

[ref-6] Bacanin N, Bezdan T, Tuba E, Strumberger I, Tuba M (2020). Optimizing convolutional neural network hyperparameters by enhanced swarm intelligence metaheuristics. Algorithms.

[ref-7] Bi J (2012). A review of statistical methods for determination of relative importance of correlated predictors and identification of drivers of consumer liking. Journal of Sensory Studies.

[ref-8] Bin J, Gardiner B, Li E, Liu Z (2020). Multi-source urban data fusion for property value assessment: a case study in philadelphia. Neurocomputing.

[ref-9] Bin J, Gardiner B, Liu Z, Li E (2019). Attention-based multi-modal fusion for improved real estate appraisal: a case study in los angeles. Multimedia Tools and Applications.

[ref-10] Can A (1992). Specification and estimation of hedonic housing price models. Regional Science and Urban Economics.

[ref-11] Chiarazzo V, Caggiani L, Marinelli M, Ottomanelli M (2014). A neural network based model for real estate price estimation considering environmental quality of property location. Transportation Research Procedia.

[ref-12] Cho H, Kim Y, Lee E, Choi D, Lee Y, Rhee W (2020). Basic enhancement strategies when using bayesian optimization for hyperparameter tuning of deep neural networks. IEEE Access.

[ref-13] Cioffi R, Travaglioni M, Piscitelli G, Petrillo A, De Felice F (2020). Artificial intelligence and machine learning applications in smart production: progress, trends, and directions. Sustainability.

[ref-14] Cui H, Bai J (2019). A new hyperparameters optimization method for convolutional neural networks. Pattern Recognition Letters.

[ref-15] Ferlan N, Bastič M, Pšunder I (2017). Influential factors on the market value of residential properties. Engineering Economics.

[ref-16] Feurer M, Hutter F (2019). Hyperparameter optimization.

[ref-17] Goldberger J, Hinton G, Roweis S, Salakhutdinov R (2005). Neighbourhood components analysis. Advances in Neural Information Processing Systems.

[ref-18] Hamzaoui YE, Perez JAH (2011). Application of artificial neural networks to predict the selling price in the real estate valuation process.

[ref-19] Han J, Choi D, Park S, Hong S (2020). Hyperparameter optimization using a genetic algorithm considering verification time in a convolutional neural network. Journal of Electrical Engineering and Technology.

[ref-20] Ho WKO, Tang B-S, Wong SW (2020). Predicting property prices with machine learning algorithms. Journal of Property Research.

[ref-21] Ho WKO, Tang B, Wong SW (2020). Predicting property prices with machine learning algorithms. Journal of Property Research.

[ref-22] Hong J, Choi H, Kim W (2020). A house price valuation based on the random forest approach: the mass appraisal of residential property in south korea. International Journal of Strategic Property Management.

[ref-23] Kang J, Lee HJ, Jeong SH, Lee HS, Oh KJ (2020). Developing a forecasting model for real estate auction prices using artificial intelligence. Sustainability.

[ref-25] Kim S, Geem ZW, Han G (2020). Hyperparameter optimization method based on harmony search algorithm to improve performance of 1D CNN human respiration pattern recognition system. Sensors.

[ref-26] Kim H, Kwon Y, Choi Y (2020). Assessing the impact of public rental housing on the housing prices in proximity: based on the regional and local level of price prediction models using long short-term memory (LSTM). Sustainability.

[ref-27] Kingma D, Ba J (2014). Adam: a method for stochastic optimization. *arXiv*.

[ref-28] Kraus M, Feuerriegel S, Oztekin A (2020). Dep learning in business analytics and operations research: models, applications and managerial implications. European Journal of Operational Research.

[ref-29] Krizhevsky A, Sutskever I, Hinton GE (2012). ImageNet classification with deep convolutional neural networks.

[ref-30] Lam KC, Yu CY, Lam KY (2008). An artificial neural network and entropy model for residential property price forecasting in Hong Kong. Journal of Property Research.

[ref-31] Lee C, Park KK (2020). Representing uncertainty in property valuation through a bayesian deep learning approach. Real Estate Management and Valuation.

[ref-32] Lima LL, Ferreira Junior JR, Oliveira MC (2020). Toward classifying small lung nodules with hyperparameter optimization of convolutional neural networks. Computational Intelligence.

[ref-33] Liu Y (2017). A commercial real estate price evaluation model based on GT-BCPSO-BP neural network. International Journal of Applied Decision Sciences.

[ref-34] Liu R, Liu Y, Yan Y, Wang J (2020). Iterative deep neighborhood: a deep learning model which involves both input data points and their neighbors. Computational Intelligence and Neuroscience.

[ref-35] Lu S, Li Z, Qin Z, Yang X, Goh RSM (2017). A hybrid regression technique for house prices prediction.

[ref-36] Madhuri CHR, Anuradha G, Pujitha MV (2019). House price prediction using regression techniques: a comparative study.

[ref-37] Manasa J, Gupta R, Narahari NS (2020). Machine learning based predicting house prices using regression techniques.

[ref-38] Milunovich G (2020). Forecasting australia’s real house price index: a comparison of time series and machine learning methods. Journal of Forecasting.

[ref-39] Mimis A, Rovolis A, Stamou M (2013). Property valuation with artificial neural network: the case of Athens. Journal of Property Research.

[ref-40] Mora-Esperanza JG (2004). Artificial intelligence applied to real estate valuation: an example for the appraisal of Madrid.

[ref-41] Morano P, Tajani F, Torre CM (2015). Artificial intelligence in property valuations: an application of artificial neural networks to housing appraisal.

[ref-42] Mu J, Wu F, Zhang A (2014). Housing value forecasting based on machine learning methods. Abstract and Applied Analysis.

[ref-43] Nejad MZ, Lu J, Behbood V (2017). Applying dynamic Bayesian tree in property sales price estimation.

[ref-44] Núñez-Tabales JM, Caridad JM, Rey FJ (2013). Artificial neural networks for predicting real estate prices. Revista de Metodos Cuantitativos para la Economia y la Empresa.

[ref-45] Peter NJ, Okagbue HI, Obasi ECM, Akinola AO (2020). Review on the application of artificial neural networks in real estate valuation. International Journal of Advanced Trends in Computer Science and Engineering.

[ref-46] Plonis D, Katkevicius A, Gurskas A, Urbanavicius V, Maskeliunas R, Damasevicius R (2020). Prediction of meander delay system parameters for internet-of-things devices using pareto-optimal artificial neural network and multiple linear regression. IEEE Access.

[ref-47] Połap D, Woźniak M, Wei W, Damaševičius R (2018). Multi-threaded learning control mechanism for neural networks. Future Generation Computer Systems.

[ref-48] Statistics Finland (2020). Statistics Finland. https://www.stat.fi/tup/paavo/paavon_aineistokuvaukset_en.html.

[ref-49] Sun Y (2019). Real estate evaluation model based on genetic algorithm optimized neural network. Data Science Journal.

[ref-50] Tajani F, Morano P, Ntalianis K (2018). Automated valuation models for real estate portfolios: a method for the value updates of the property assets. Journal of Property Investment Finance.

[ref-51] The Ministry of the Environment and the Housing Finance and Development Centre of Finland (2020). Hintatiedot. https://asuntojen.hintatiedot.fi/haku/?l=2&submit=In+English.

[ref-52] Varma A, Sarma A, Doshi S, Nair R (2019). House price prediction using machine learning and neural networks.

[ref-53] Wang W, Chang Y, Wang H (2019). An application of the spatial autocorrelation method on the change of real estate prices in taitung city. ISPRS International Journal of Geo-Information.

[ref-54] Wang Y, Li Y, Song Y, Rong X (2020). The influence of the activation function in a convolution neural network model of facial expression recognition. Applied Sciences.

[ref-55] Weights & Biases (2020). Weights & biases. https://www.wandb.com/.

[ref-56] Xu H, Gade A (2017). Smart real estate assessments using structured deep neural networks.

[ref-57] Xue C, Ju Y, Li S, Zhou Q, Liu Q (2020). Research on accurate house price analysis by using GIS technology and transport accessibility: a case study of Xi’an, China. Symmetry.

[ref-58] Zhang M, Jing W, Lin J, Fang N, Wei W, Woźniak M, Damaševičius R (2020). NAS-HRIS: automatic design and architecture search of neural network for semantic segmentation in remote sensing images. Sensors.

[ref-59] Zhang Q, Zhu S (2018). Visual interpretability for deep learning: a survey. Frontiers of Information Technology Electronic Engineering.

[ref-60] Zhao Y, Chetty G, Tran D (2019). Deep learning with XGBoost for real estate appraisal.

[ref-61] Zhou X (2020). The usage of artificial intelligence in the commodity house price evaluation model. Journal of Ambient Intelligence and Humanized Computing.

[ref-62] Štubňová M, Urbaníková M, Hudáková J, Papcunová V (2020). Estimation of residential property market price: comparison of artificial neural networks and hedonic pricing model. Emerging Science Journal.

